# Elevated plasma levels of endothelin are associated with the severity of sepsis and presence of shock in contrast to the levels of atrial natriuretic peptide

**DOI:** 10.1155/S0962935192000632

**Published:** 1992

**Authors:** A. Beishuizen, J. M. Götz, L. Kip, C. Haanen, I. Vermes

**Affiliations:** 1 Department of Internal Medicine Medical Spectrum Twente P.O. Box 50000 Enschede 7500 KA The Netherlands; 2 Department of Clinical Chemistry Medical Spectrum Twente P.O. Box 50000 Enschede 7500 KA The Netherlands

## Abstract

Immunoreactive endothelin (ETi) and atrial natriuretic peptide (ANPi) blood levels were measured by radioimmunoassay in patients with clinically defined sepsis. The interaction between these two peptides and their relation to circulatory shock and mortality were studied. All septic patients (n = 16) had significantly higher ETi (22.3 ± 11.1 pg/ml) and ANPi (398.3 ± 154.3 pg/ml) plasma concentrations compared to control subjects (ETi, 4.1 ± 1.2; ANPi, 59.1 ± 14.8 pg/ml; n = 13). ETi levels followed the severity of illness according to the APACHE II scoring system and were higher in patients who did not survive. ETi levels were significantly higher in the presence of shock and bacteraemia. Furthermore, ETi correlated well with plasma lactate (r = 0.83, p < 0.05), but not with renal function. ANPi levels did not show correlation with any of these determinants. Serial blood sampling, six consecutive days after admission, showed that ETi levels gradually decreased in normotensive patients in contrast to patients with septic shock. ANPi levels did not show systematic changes in time, and no relationship was observed between ETi and ANPi levels. These results suggest that plasma ETi levels are indicative for disease severity and might have prognostic significance. The role of ANPi during sepsis remains to be eludicated.


Mediators of Inflammation 1, 419–423 (1992)

### Elevated plasma levels of endothelin are associated with the severity of sepsis and presence of shock in contrast to the levels of atrial natriuretic peptide

A. Beishuizen,[1] J. M. Götz,[1] L. Kip,[2] C. Haanen,[2] and I. Vermes[2,CA]

[1]Department of Internal Medicine, and [2]Department of Clinical Chemistry, Medical Spectrum Twente, P.O. Box 50000, 7500 KA Enschede, The Netherlands

[CA] Corresponding Author

**IMMUNOREACTIVE endothelin (ETi) and atrial natriuretic peptide (ANPi) blood levels were measured by radioimmunoassay in patients with clinically defined sepsis. The interaction between these two peptides and their relation to circulatory shock and mortality were studied. All septic patients ($n = 16$) had significantly higher ETi (22.3 ± 11.1 pg/ml) and ANPi (398.3 ± 154.3 pg/ml) plasma concentrations compared to control subjects (ETi, 4.1 ± 1.2; ANPi, 59.1 ± 14.8 pg/ml; $n = 13$). ETi levels followed the severity of illness according to the APACHE II scoring system and were higher in patients who did not survive. ETi levels were significantly higher in the presence of shock and bacteraemia. Furthermore, ETi correlated well with plasma lactate ($r = 0.83$, $p < 0.05$), but not with renal function. ANPi levels did not show correlation with any of these determinants. Serial blood sampling, six consecutive days after admission, showed that ETi levels gradually decreased in normotensive patients in contrast to patients with septic shock. ANPi levels did not show systematic changes in time, and no relationship was observed between ETi and ANPi levels. These results suggest that plasma ETi levels are indicative for disease severity and might have prognostic significance. The role of ANPi during sepsis remains to be eludicated.**

**Key words:** Atrial natriuretic peptide, Endothelin, Sepsis, Septic shock, Severity of illness index

### Introduction

Sepsis is a life threatening syndrome, mostly induced by bacterial infections, which may be complicated by shock, multiple organ failure, and which carries a high mortality.[1] The severity of the disease is generally believed to be aggravated by the release and activation of endogenous mediators in response to bacterial products, like endotoxins.[1] An imbalance between vasodilating and vasoconstrictive factors may result in systemic vasodilatation, and selective vasoconstriction in circumscript vascular beds, as in the kidney.

Since it has become evident that endothelium cells are capable of releasing vasoactive substances that regulate vascular smooth muscle tone, these substances have raised growing interest with regard to the pathogenesis of the cardiovascular changes induced by septic shock.[1,2] It is well documented that endothelin (ET), a peptide produced and secreted by the endothelium, has extremely potent vasoconstrictor and vasopressor actions and decreases cardiac output and renal blood flow.[3,4] Recent studies showed a five- to ten-fold increase of ET in the sepsis syndrome.[5,6] Atrial natriuretic peptide (ANP), which is released by the cardiac atria in response to a rise in blood pressure and volume, induces a fall in blood pressure and a rise in renal blood flow.[7] In this respect ANP is a potential counter-reacting mediator of ET.[7] ET has been reported to directly or indirectly enhance the release of ANP, while increased ANP release may act to preserve the effects of ET on cardiovascular and renal functions.[8,9]

Theoretically, an imbalance in the release of these two vasoactive peptides could induce or aggravate the cardiovascular and renal changes characteristic for sepsis. The aim of the present study was to test this hypothesis. The profile of ET and ANP was examined with regard to the severity of disease, mortality and the presence of shock in patients with sepsis. Also the relationship between these vasoactive hormones and other determinants like renal function and plasma lactate was studied.

### Materials and Methods

*Subjects:* This study was performed in a total of 29 subject, with the approval of the hospital Medical Ethics Committee.

Sixteen consecutive patients (ten female, six male; mean age 72 years) with severe sepsis were studied. The patients were admitted to the Intensive Care Unit of our hospital during 1992. The criteria for the diagnosis of sepsis were: hypothermia

© 1992 Rapid Communications of Oxford Ltd

(< 35.6°C) or fever (> 38.3°C); tachycardia (> 90 beats/min) and tachypnoea (> 20 breaths/min or the requirement of mechanical ventilation), as well as two of the following six signs of inadequate organ function or perfusion: unexplained metabolic acidosis (pH < 7.3, base deficit of > 5 mmol/l, or an elevated lactate level); hypoxaemia ($pO_2$ < 10 kPa or ratio of the partial pressure of oxygen to the fraction of inspired oxygen < 250); acute renal failure (urinary output < 0.5 ml/kg/h); coagulation abnormalities; acute alteration in mental status; and cardiac index of more than 4 $l.min^{-1}.\ m^{-2}$ of body surface area with systemic vascular resistance of less than 800 $dyn.s.cm^{-5}$.[10] Septic shock was defined as sepsis with hypotension (systolic BP < 90 mmHg or a reduction of > 40 mmHg from baseline in the absence of other causes for hypotension), despite adequate fluid suppletion along with the presence of perfusion abnormalities.[10]

In the septic patients, severity of illness on admission was scored according to the Acute Physiology and Chronic Health Evaluation (APACHE II) scoring system.[11] Systemic dynamics were studied using a pulmonary artery catheter ($n$ = 10). Patients were studied during the early phase of sepsis/septic shock to minimize the influence of therapeutic interventions on haemodynamic and metabolic values.

The control group included 13 age- and sex-matched healthy subjects. Patients with essential hypertension, congestive heart failure, chronic renal failure or liver cirrhosis were excluded from this study.

*Blood samples:* On admission and with 8 h intervals on day 1 and 2, and once daily on days 3 to 6, blood samples were drawn by venipuncture or from an indwelling venous catheter. Blood was collected into pre-chilled tubes containing EDTA. Specimens were immediately centrifuged at 4°C and the plasma was transferred to plastic tubes containing a kallikrein inhibitor, aprotinin (200 KiU/ml blood, Trasylol, Bayer, Leverkusen, Germany) and stored at −15°C. Blood samples of the control subjects were collected between 08.00 and 10.00 h in the supine position after 30 min rest.

*Methods:* Immunoreactive endothelin (ETi) concentrations were measured with a commercially available radioimmunoassay (Nichols Institute, San Juan Capistrano, CA, USA) after purification and concentration of the plasma samples. Acidified plasma samples (2 ml) were extracted by Sep Pak, C18 reverse phase cartridges (Millipore Corp., Millford, MA, USA) and eluted with ethanol. Each sample was evaporated to dryness under a stream of nitrogen and the residue dissolved in assay buffer and measured in duplicate. The results are expressed as pg/ml ETi using endothelin-1 as standard. On the molar basis, the antiserum showed 100% crossreactivity with endothelin-1, 52% with endothelin-2, 96% with endothelin-3, 7% with big-endothelin and < 0.1% with other peptides such as ANP, vasopressin or angiotensin II. The detection limit of the assay (2 SD of the zero-point binding) was 1 pg/ml ($n$ = 20). The interassay and intra-assay coefficients of variation were 6.8% ($n$ = 12) and 4.5% ($n$ = 20), respectively. The normal value for ETi is 2.4–5.4 pg/ml.

Plasma immunoreactive ANP (ANPi) concentrations were measured with a radioimmunoassay (Nichols Institute, San Juan Capistrano, CA, USA). Acidified plasma samples (0.5 ml) were extracted on Sep Pak chromatography column, and measured in duplicate. Results are expressed in pg/ml ANPi using human alpha-ANP as standard. The antiserum has 100% crossreactivity with human alpha-ANP, ANP 5–28 and ANP 7–28 calculated on the molar basis and no crossreaction with MSH, vasopressin, ACTH, endothelin and other ANP fragments such as ANP 13–28 or ANP 1–11. The detection limit of the assay was 7.5 pg/ml. The interassay and intra-assay coefficients of variation were 14.5% ($n$ = 8) and 7.7% ($n$ = 12), respectively. The normal value for ANPi is 30–90 pg/ml.

24-h urine was collected for 6 days for determination of creatinine clearance.

*Statistical analysis:* Values are expressed as mean ± SD. Linear regression analysis was used to determine correlations between plasma ETi and ANPi concentrations and other variables. Statistical analysis was performed by Student's $t$-test; $p < 0.05$ was considered a significant difference.

### Results

A group of 16 patients with clinically defined sepsis was studied. A shock state, as defined above, was confirmed in six patients. Median APACHE II score on admission was 28 and ranged from 18 to 47. Bacteraemia was confirmed by blood cultures in seven patients. Six patients died, of whom four died within 48 h after admission. The comparison between survivors and nonsurvivors is shown in Table 1. As expected, the APACHE II score in patients who died was higher than in survivors. The survivors showed lactate levels three times lower than the nonsurvivors (2.7 mmol/l *vs.* 9.9 mmol/l, $p < 0.01$). On admission, all septic patients had increased ETi and ANPi concentrations compared to age- and sex-matched controls, mean values 22.3 ± 11.1 pg/ml and 398.3 ± 154.3 pg/ml, respectively (Table 2). The ETi levels followed the severity of illness and were higher in patients who died than in survivors: 28.6 ± 13.3 *vs.*

**Table 1.** Data from the 16 patients according to survival and mortality

| | Survivors | Nonsurvivors |
|---|---|---|
| Number | 10 | 6 |
| Mean age (years) | 71.5 ± 13 | 68 ± 24 |
| Bacteraemia | 5 | 2 |
| Shock | 2 | 4 |
| APACHE II | 24 ± 7 | 31 ± 9* |
| Plasma lactate (mmol/l) | 2.7 ± 2 | 9.9 ± 9* |
| Plasma creatinine (μmol/l) | 214 ± 84 | 232 ± 135 |
| ETi level (pg/ml) | 18.5 ± 8.0 | 28.6 ± 13.3* |
| ANPi level (pg/ml) | 419.6 ± 114.2 | 362.8 ± 213.4 |

Values represent the mean ± SD. Statistical significance by Student's *t*-test: *$p < 0.05$ survivors *vs.* nonsurvivors.

**Table 2.** Immunoreactive endothelin (ETi) and atrial natriuretic peptide (ANPi) plasma concentrations in patients with severe sepsis and age- and sex-matched control subjects in relation to shock and mortality

| | *n* | ETi (pg/ml) | ANPi (pg/ml) |
|---|---|---|---|
| Controls | 13 | 4.1 ± 1.2 | 59.1 ± 14.8 |
| All patients | 16 | 22.3 ± 11.1* | 398.3 ± 154.3* |
| Sepsis | 10 | 14.1 ± 2.9 | 366.1 ± 122.0 |
| Septic shock | 6 | 27.3 ± 11.3** | 417.7 ± 174.1 |
| Survivors | 10 | 18.5 ± 8.0 | 419.6 ± 114.2 |
| Nonsurvivors | 6 | 28.6 ± 13.3*** | 362.8 ± 213.4 |

Values represent the mean ± SD of the highest concentrations measured during the first 48 h after admission. Statistical significance by Student's *t*-test $p < 0.05$: *all patients *vs.* controls; **septic patients without shock *vs.* those with shock; ***survivors *vs.* nonsurvivors. *n* = number of subjects.

18.5 ± 8 pg/ml. In our patients, there was a significant linear correlation between ETi levels on admission and the APACHE II score ($r = 0.57$, $p < 0.05$). Furthermore, linear regression demonstrated a significant correlation between ETi and plasma lactate levels ($r = 0.83$, $p < 0.05$). No correlation between ETi levels and serum creatinine or creatinine clearance was found. In contrast, ANPi levels did not differ significantly between the various subgroups (Table 2), and no correlation was found between ANPi blood levels and other determinants.

ETi levels were significantly higher in the presence of shock with positive bloodcultures, whereas the plasma ANPi levels were not different when related to the presence or absence of shock and bacteraemia (Table 3).

ETi levels in patients with septic shock appeared to be significantly ($p < 0.01$) higher than in normotensive septic patients, again in contrast to ANPi levels. Furthermore, we also found a negative correlation between ETi and mean arterial pressure ($r = -0.47$, $p < 0.05$).

In eleven patients we tested serial samples during a period of six consecutive days after admission. The evolution of ETi plasma levels over time is shown in Fig. 1. In the group of patients with septic shock ETi levels remained stable in most patients, in contrast to the group of normotensive septic patients in which a significant decrease was noted (day 1, 13.9 ± 3.1 pg/ml *vs.* day 6, 8.3 ± 2.3 pg/ml, $p < 0.05$). When looked upon longitudinally ANPi levels showed no trend or change (results not shown).

### Discussion

The present study shows that patients with sepsis exhibit at the moment of admission markedly increased plasma levels of ETi and that the height of these levels is correlated with the severity of the illness, according to the APACHE II scoring system.[11] Moreover, nonsurvivors had higher ETi levels than survivors. These data are in agreement with the findings of Pittet *et al.*[5] in eleven patients and of Weitzberg *et al.*[6] in six patients with sepsis. Recent animal studies showed also an increase of ETi during endotoxaemia.[12–14] ET is a ten-fold more potent vasoconstrictor than angiotensin II and has extremely long-lasting pressor effects.[4] Severe sepsis is characterized by an increase in cardiac output, general vasodilatation and/or hypotension. These cardiovascular changes are mediated by endogenous vasodilatators like nitric oxide and ANP and vasoconstrictors like ET. A close interaction is present between these vasoactive

**Table 3.** Immunoreactive endotheline (ETi) and atrial natriuretic peptide (ANPi) plasma concentrations in septic patients according to the presence or absence of bacteraemia and shock

| Bacteraemia | Shock | *n* | APACHE II | ETI (pg/ml) | ANPi (pg/ml) |
|---|---|---|---|---|---|
| − | − | 2(0) | 21 ± 4 | 15.3 ± 2.1 | 358 ± 200 |
| + | − | 4(0) | 25 ± 6 | 14.3 ± 3.1 | 370 ± 107 |
| − | + | 7(4) | 31 ± 6 | 20.7 ± 3.7 | 436 ± 167 |
| + | + | 3(2) | 35 ± 10 | 43.7 ± 6.1* | 373 ± 219 |

Values represent the mean ± SD of the highest ETi and ANPi concentrations during the first 48 h after admission. Statistical significance by Student's *t*-test $p < 0.05$: *septic shock patients with bacteraemia *vs.* those without bacteraemia. *n* = number of patients, deaths in parentheses.

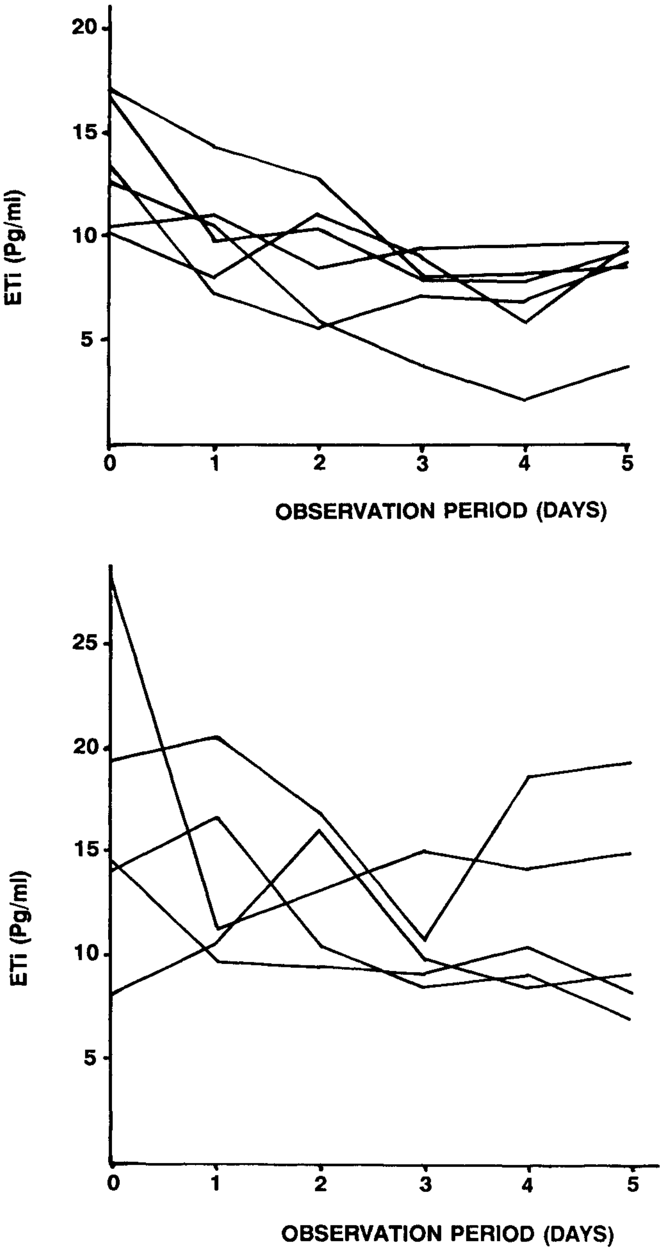

FIG. 1. Evolution of immunoreactive endothelin (ETi) plasma levels in time in normotensive septic patients (upper panel) and in patients with septic shock (lower panel).

substances; ET stimulates nitric oxide production,[15] and therefore possibly contributing to the vascular hyporesponsiveness found in septic shock,[16] while nitric oxide inhibits ET release.[17] Despite these changes, plasma ETi levels are increased in septic patients, suggesting that ETi possibly acts as a local regulatory peptide leading to (selective) vasoconstriction. Moreover, ETi levels correlated well with symptoms of shock, and there was an inverse relationship with mean arterial pressure. Possibly, ETi is no longer effective in controlling systemic arterial pressure under these pathological conditions. We also found a significant correlation between ETi and plasma lactate levels, reflecting the severity of the shock state. No relation was found between ETi and renal function, excluding renal failure as the cause for the increased ETi levels observed in our patients.[18]

To the authors' knowledge no data exist about the plasma levels of ANPi in patients with sepsis. Eison *et al.*[19] observed an increase of ANPi in twelve patients with adult respiratory distress syndrome. A preliminary study of Mitaka *et al.*[20] reported similar findings in patients with respiratory failure associated with sepsis. In an ovine model of endotoxaemia a 13-fold increase or ANPi was found, leading to increased diuresis and natriuresis.[21]

ET has been reported to enhance ANP release and because ANP by its vasodilatory and natriuretic properties counteracts ET, we expected increased ANPi levels in patients with sepsis.[8,9] Our findings were in accordance with this supposition, as plasma levels of ANPi appeared significantly elevated in all 16 patients. However, in contrast to ETi, the ANPi levels showed no relation with the severity of the sepsis, neither with the presence of shock.

It is generally accepted that ET elevation is due to damage of the endothelium,[3,4] the rise of ANPi may be an expression of many different trigger mechanisms like atrial stretch, changes in intravascular volume, tachycardia, adrenal and pituitary gland hormones.[7] The complete lack of correlation between ETi levels and ANPi levels exclude ET stimulation as the principal cause of increased ANPi in patients with sepsis.

The prolonged elevation of ETi we observed during 6 consecutive days poses the question of whether this is the result of stimulated continuous synthesis or if it is a reflection of reduced elimination of ET. The observed longstanding high ETi levels, especially in patients with septic shock, certainly play an essential part in the evolution and severity of this disease.

In conclusion, the present observations demonstrate that ETi concentrations are correlated with the severity and final outcome of sepsis and that ANPi concentrations are not. ETi levels are associated with the presence of a shock state and with the degree of tissue ischaemia reflected by plasma lactate concentrations. Further clinical studies are needed to clarify the precise role of ET in the pathogenesis and course of sepsis.

### References

1. Bone RC. The pathogenesis of sepsis. *Ann Intern Med* 1991; **115**: 457–469.
2. Billiau A, Vandekerckhove F. Cytokines and their interactions with other inflammatory mediators in the pathogenesis of sepsis and septic shock. *Eur J Clin Invest* 1991; **21**: 559–573.
3. Vane JR, Anggard EE, Botting RM. Regulatory functions of the vascular endothelium. *N Engl J Med* 1990; **323**: 27–36.
4. Doherty AM. Endothelin; a new challenge. *J Med Chem* 1992; **35**: 1493–1508.

5. Pittet JF, Morel DR, Hemsen A, *et al.* Elevated plasma endothelin-1 concentrations are associated with the severity of illness in patients with sepsis. *Ann Surg* 1991; **213**: 261–264.
6. Weitzberg E, Lundberg JM, Rudehill A. Elevated plasma levels of endothelin in patients with sepsis syndrome. *Circ Shock* 1991; **33**: 222–227.
7. Goetz KL. Physiology and pathophysiology of atrial peptides. *Am J Physiol* 1988; **254**: E1–E15.
8. Goetz KL, Wang BC, Madwed JB, Zhu JL, Leadley Jr. RJ. Cardiovascular, renal, and endocrine responses to intravenous endothelin in conscious dogs. *Am J Physiol* 1988; **255**: R1064–R1068.
9. Ota K, Kimura T, Shoji M, *et al.* Interaction of ANP with endothelin on cardiovascular, renal, and endocrine function. *Am J Physiol* 1992; **262**: E135–E141.
10. American College of Chest Physicians/Society of Critical Care Medicine Consensus Conference. Definitions for sepsis and organ failure and guidelines for the use of innovative therapies in sepsis. *Crit Care Med* 1992; **20**: 864–874.
11. Knaus WA, Draper EA, Wagner DP, Zimmerman JE. APACHE II: A severity of disease classification system. *Crit Care Med* 1985; **13**: 818–829.
12. Pernow J, Hemsen A, Lundberg JM. Increased plasma levels of endothelin-like immunoreactivity during endotoxin administration in the pig. *Acta Physiol Scand* 1989; **137**: 317–318.
13. Morel DR, Lacroix JS, Hemsen A, Steinig DA, Pittet JF, Lundberg JM. Increased plasma and pulmonary lymph levels of endothelin during endotoxin shock. *Eur J Pharmacol* 1989; **167**: 427–428.
14. Sigiura M, Inagami T, Kon V. Endotoxin stimulates endothelin-release *in vivo* and *in vitro* as determined by radioimmunoassay. *Biochem Biophys Res Comm* 1989; **161**: 1220–1227.
15. Emori T, Hirata Y, Kanno K, *et al.* Endothelin-3 stimulates production of endothelium-derived nitric oxide via phosphoinositide breakdown. *Biochem Biophys Res Comm* 1991; **174**: 228–235.
16. Kilbourn RG, Jubran A, Gross SS, *et al.* Reversal of endotoxin-mediated by NG–methyl–L–arginine, an inhibitor of nitric oxide synthesis. *Biochem Biophys Res Comm* 1990; **172**: 1132–1138.
17. Boulanger C, Luscher TF. Release of endothelin from the porcine aorta. Inhibition by endothelium-derived nitric oxide. *J Clin Invest* 1990; **85**: 587–590.
18. Simonson MS, Dunn MJ. Endothelin peptides: A possible role in glomerular inflammation. *Lab Invest* 1991; **64**: 1–4.
19. Eison HB, Rosen MJ, Phillips RA, Krakoff LR. Determinants of atrial natriuretic factor in the adult respiratory distress syndrome. *Chest* 1988; **94**: 1040–1045.
20. Mitaka C, Nagura T, Sakanishi N, Tsunoda Y, Toyooka H. Plasma alpha-atrial natriuretic peptide concentrations in acute respiratory failure associated with sepsis: preliminary study. *Crit Care Med* 1990; **18**: 1201–1203.
21. Lubbesmeyer HJ, Woodson L, Traber LD, Flynn JT, Herndon DN, Traber DL. Immunoreactive atrial natriuretic factor is increased in ovine model of endotoxemia. *Am J Physiol* 1988; **254**: R567–R571.

**Received 8 September 1992; accepted in revised form 30 September 1992**
